# The NBS Large-Area Alpha-Particle Counting Systems

**DOI:** 10.6028/jres.092.031

**Published:** 1987-10-01

**Authors:** J. M. R. Hutchinson, S. J. Bright

**Affiliations:** National Bureau of Standards, Gaithersburg, MD 20899

**Keywords:** alpha particle, large area, monitoring, proportional counting, radioactivity, standards

## Abstract

Two alpha-particle counting systems for the measurement of large-area sources have been developed at the National Bureau of Standards. The systems and their characteristics are described. One system uses an internal-source proportional counter and the other measures sources external to the counting volume through a thin aluminized mylar window. The “internal” system is used to measure sources in the lower activity ranges. These calibrated sources arc then used to establish the efficiency of the “external” counter used to measure the higher-activity sources.

## 1. Introduction

Recently the National Bureau of Standards (NBS) has developed two alpha-particle counting systems for the calibration of large area sources. These internal-source and an external-source gasproportional counting systems were requested by the United States Air Force (USAF) to calibrate large-area sources to serve as transfer standards between the USAF and NBS. The sources are rectangular, 8-in × 5-in, with ^238^Pu deposited on an aluminum substrate. The active area is an array of 1-mm diameter dots spaced a minimum of 4 mm apart. The four sources used range in total activity from 10^2^ to 10^5^ Bq.

Measurements described here have characterized the potential errors when calibrations are performed with the two systems, and when the calibrated sources are used to calibrate field monitoring equipment.

## 2. Counting Systems

### 2.1 Internal Gas-Proportional Counter

**Figure 1:**
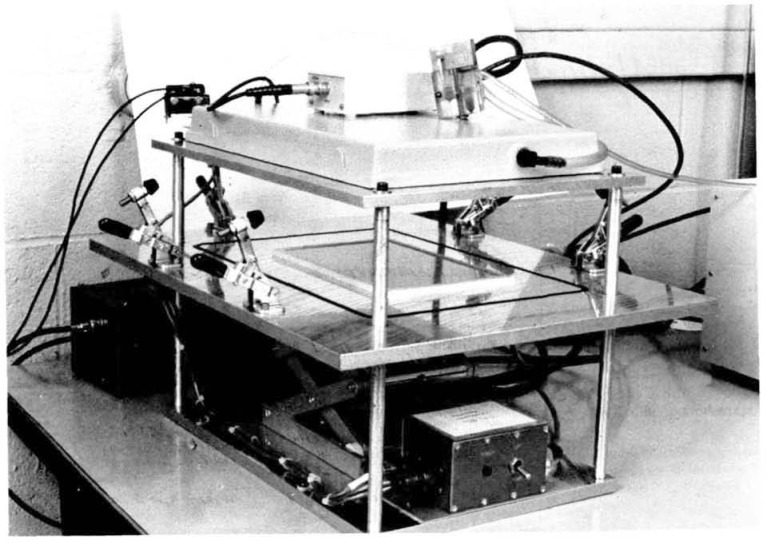
Internal 2*π*-alpha-particle gas-proportional counter with a large-area source in counting position. When counting, the baseplate is raised and the clamps are tightened.

**Figure 2:**
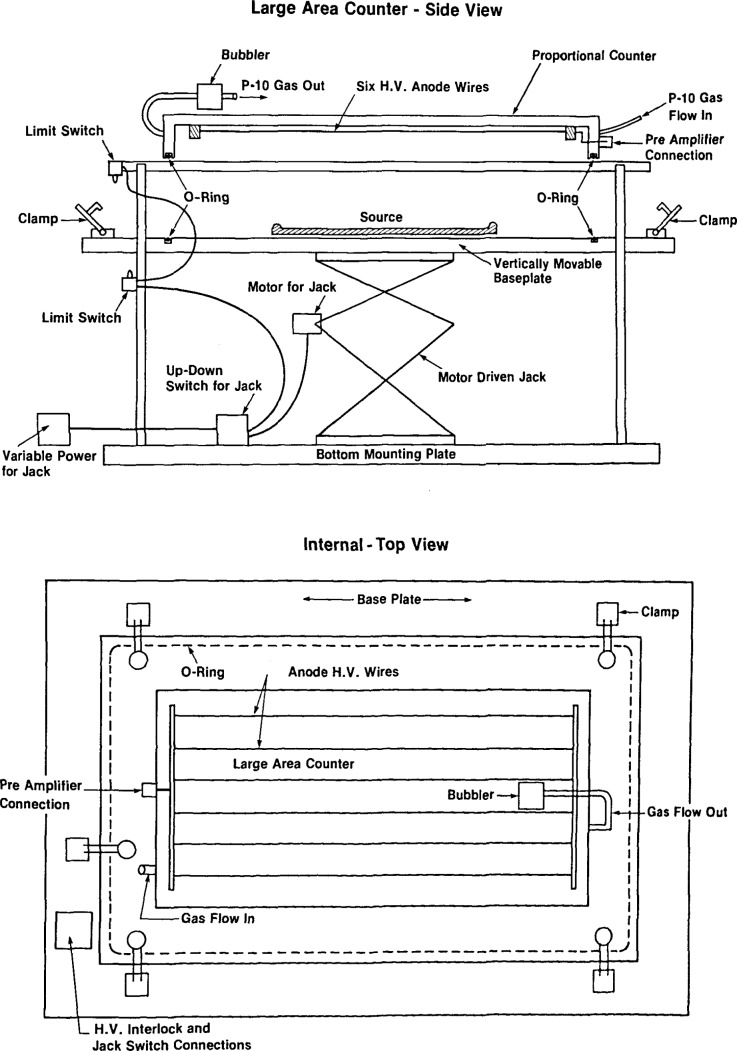
**a and b**-Schematics of the internal gas-proportional counter. The detector has 19 anode wires with 1 cm separation (fig. 2b)

The counter is pictured in figure 1 and shown schematically in figures 2a and 2b. A 13-in × 9-in Herfurth large-area flow proportional counter HGZ 730-C is mounted on four aluminum pedestals. The various grills, safety grids, and aluminized-mylar-foil window are removed and replaced with a vertically movable baseplate which supports the source and seals the counting volume by means of an “O” ring. Pressure for the seal is applied by five clamps. A clamp was not mounted on one of the sides, thereby permitting easy insertion of the source on this side. The baseplate is moved up and down with a lab-jack which is driven by a small motor. The vertical excursions both at the top and bottom of the baseplate movement are limited by microswitches which cut off the motor when the baseplate presses on them. The baseplate is not attached to the lab-jack so that if the motorized downward motion of the jack were accidently activated while the clamps were closed, the baseplate would remain clamped to the rest of the counter, the jack would move down alone, and no damage would result to the motor.

The normal counting gas is P-10 at atmospheric pressure. Typically it takes 2–3 minutes after the gas flow is turned on before the alpha-particle pulses reach full height. After that, the counting space is flushed at a rate of about a bubble through the bubbler per one or two seconds. The procedure for counting a source in this counter is given in the Appendix.

**Figure 3:**
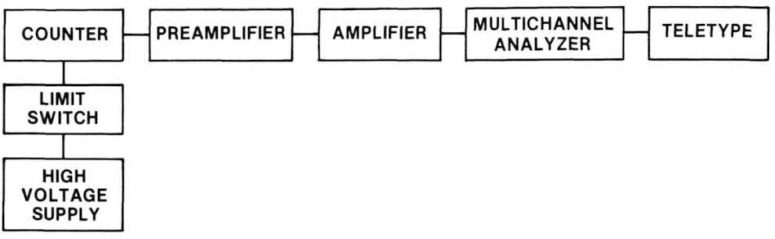
Schematic diagram of the electronics for both internal and external counters.

A schematic drawing of the electronics for this and the external counting system is given in figure 3. Pulses originating in the counting volume as a result of energetic alpha particles producing ions within the counter, with subsequent gas multiplication, are passed into a charge-sensitive preamplifier, then into an amplifier, and are recorded in a multichannel analyzer which is read out on tape for subsequent evaluation. The pulse-height spectrum can be monitored on the MCA screen.

The high voltage is applied through a circuit which cuts off whenever the upper limiting microswitch is not under pressure from the baseplate. This is a safety feature which makes it impossible for an operator to introduce a source into the counter while the counter wires are at high voltage.

### 2.2 ‘External’ Gas Proportional Counter

**Figure 4:**
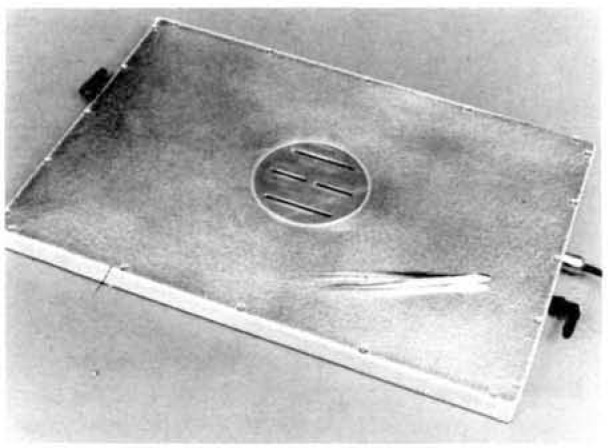
External-defined-aperture, thin-window, alpha-particle, gas-proportional counter with the slit baffle in place. The tweezers are used to remove the baffle carefully, so that the delicate mylar window is not damaged.

**Figure 5:**
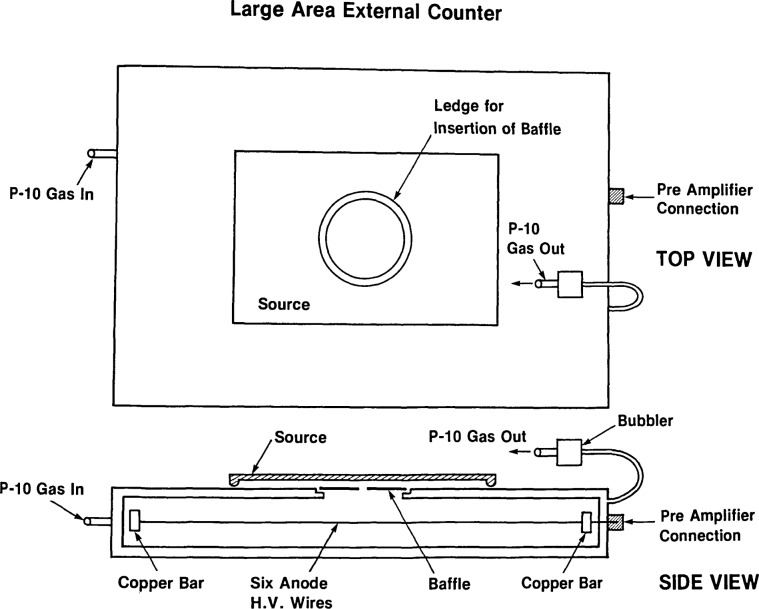
Schematic diagram of the external counter.

The counter is pictured in figure 4 and shown schematically in figure 5. It is used to compare sources with activities too high for the internal counter with previously calibrated standards. This counter is of identical original design to the internal counter. However, the movable “baseplate” is replaced by an aluminized mylar window which is supported by a mounting plate, permanently attached. Baffles can be inserted to reduce count rates to acceptable levels for very active sources.

The large-area source is inverted and placed onto the mounting plate and this represents the counting position. In order to make accurate comparisons with standards in this counting geometry, it was necessary that the source-to-detector configurations be reproducible and also that the active layer be “thin” to the emitted alpha particles so that variations in distance from the counter would not affect the count rate significantly.

The expectation is that this source would provide a calibration device for essentially monoenergetic alpha particles for the monitoring instrument. The readout in a field measurement of surface contamination would be related to this calibration through a previously determined factor which would take into account alpha-particle absorption in the source These factors are being developed into a forthcoming ISO standard.

## 3. Large-Area Sources

**Figure 6:**
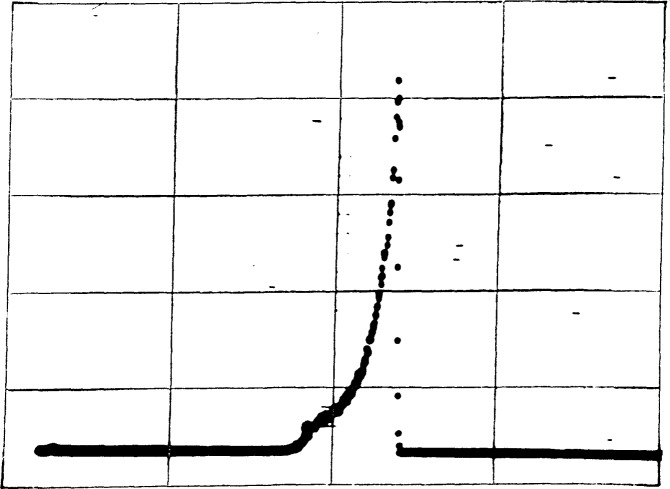
Silicon surface-barrier spectrum of the ^238^Pu large-area sources. This shows that the peak to be well resolved, an important requirement in reducing systematic error in the calibration.

With this approach in mind, high-specific-activity ^238^Pu rather than the relatively massive Pu was chosen as the source nuclide. With this choice, sources could be provided in which the emitted alpha-particle peak taken, for example, with a surface barrier detector is clearly resolved (fig. 6).

**Figure 7:**
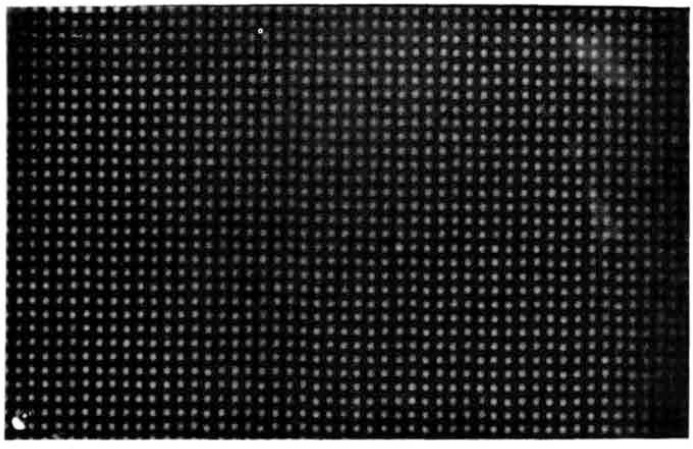
Radiograph of ^238^Pu large-area source number AA373 full scale.

The sources were deposited as an array of quantitative drops on an aluminum substrate and affixed by an anodizing process. A full scale radiograph of one of the sources is shown in figure 7. The active material is quite stable. For example, the results of a series of “swipe” tests show very little active material to be removed (table 1). Two sets of four sources ranging in total activity from 10^2^ to 10^5^ Bq were provided, one set to be retained by the USAF and one set to be kept at NBS.

The source size, easily accepted by the internal counter, was chosen because it was large enough to calibrate the present USAF monitoring instruments. AN/PDR-56F. Currently, the USAF calibrates these instruments with the AN/UDM-7C source sets. The new NBS large-area sources were designed to be compatible with retrofitting into the existing AN/UDM-7C jig in place of the existing sources which the USAF desires to replace. If the USAF should replace the AN/PDR-56F with another instrument in the future, the NBS large-area source design should be adaptable for that eventuality.

## 4. Tests of the Counters

**Figure 8:**
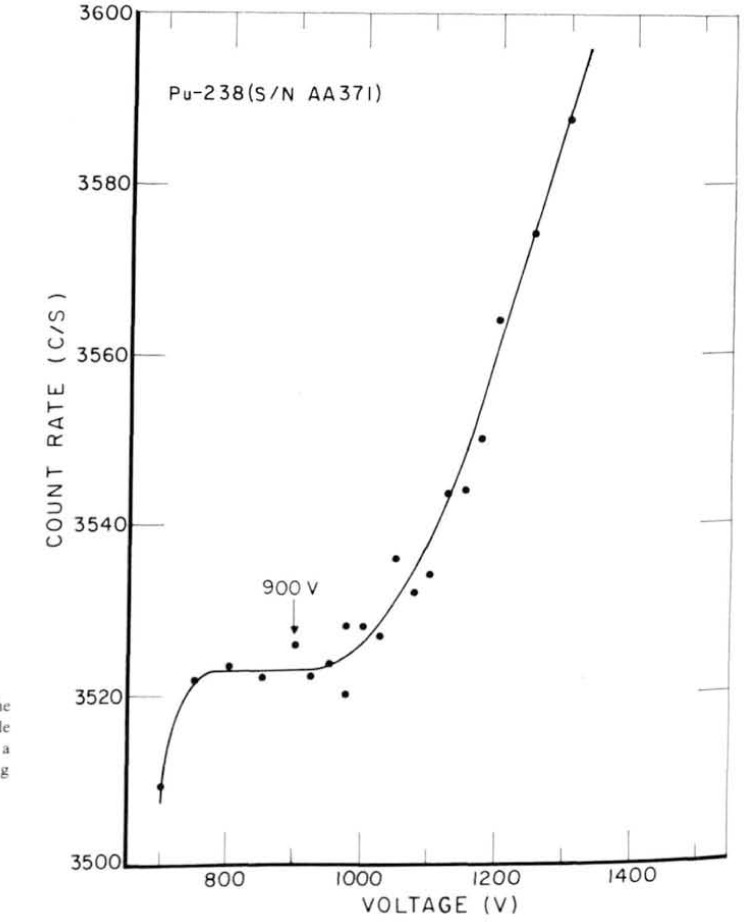
Voltage plateau of the large-area 2*π*-alpha-particle proportional counter using a large-area source (Operating voltage was set at 900 V.)

**Figure 9:**
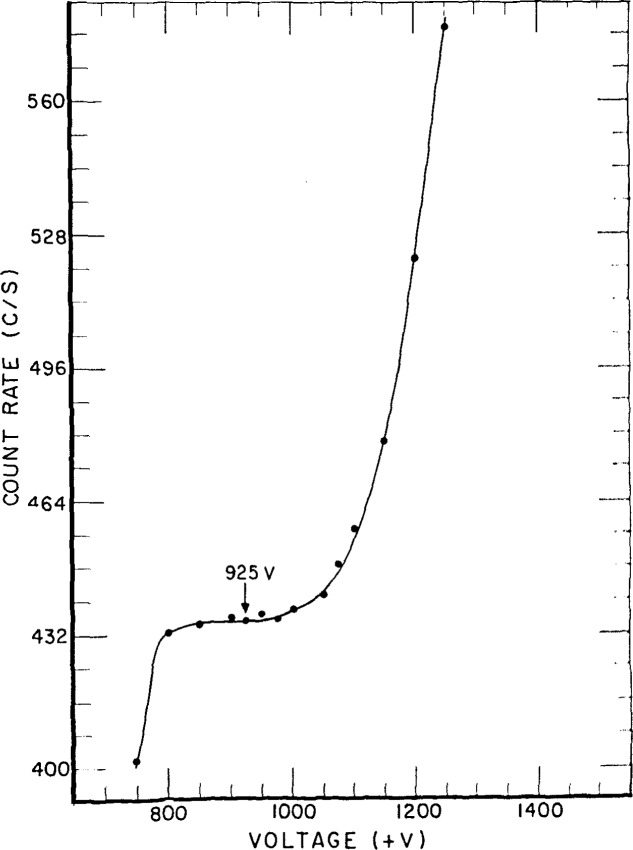
Voltage plateau of the external counter for a large-area ^238^Pu source. (Operating voltage was set at 925 V.)

Figures 8 and 9 show the high voltage plateau for the internal and external counters, respectively. Clearly the adaptation from the external mode to internal produces little change in its functioning.

**Figure 10:**
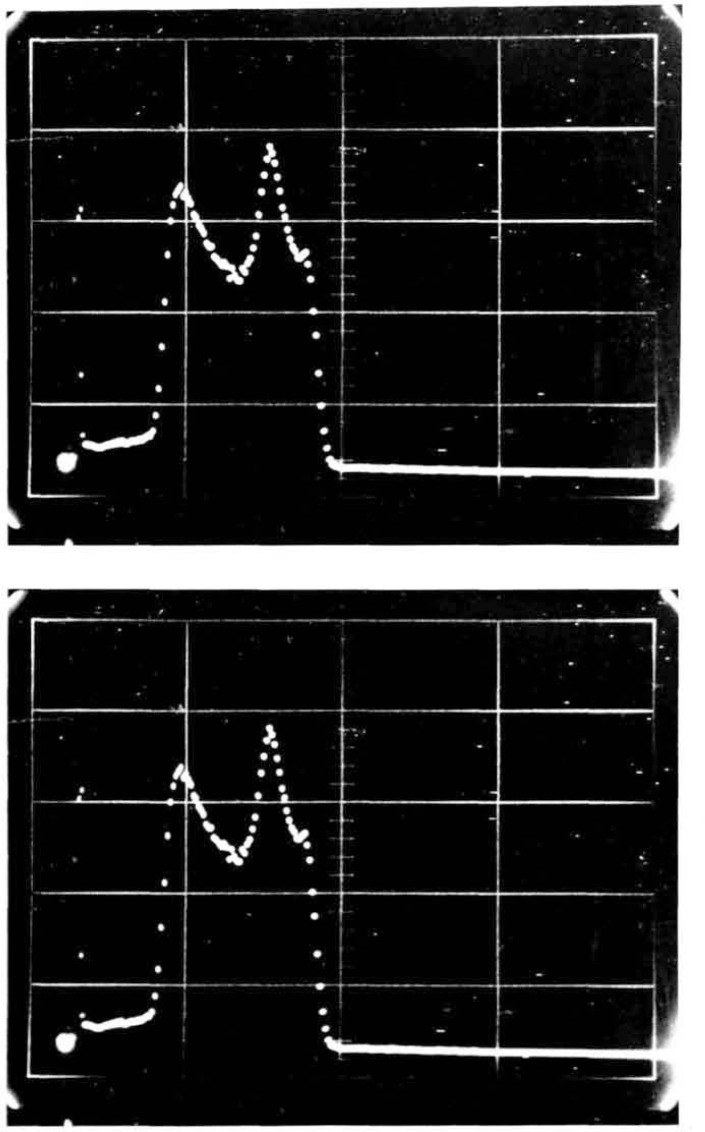
**a and b**-Pulse-height spectra for the external counter for a large-area source using a 5-cm aperture (10a) and for a point source (10b).

Figures 10a and 10b show the pulse-height spectra for the external counter for a 5 cm aperture for a large area source and for a point source. The two peaks do not represent different alpha-particle groups but rather result from different degrees of gas multiplication in different parts of the detector. Although we have not performed a thorough investigation of the effect, the initial direction of the alpha-particle relative to the anode wires affects the pulse height.

## 5. Homogeneity Tests Of the Superficial Activity Of the Sources

It is important that the activity deposits be homogeneously distributed over the source. The instrument reads, and will be calibrated, in terms of activity per square centimeter. The calibration is performed by placing the instrument over some part of the source and recording the readout which then represents the superficial activity value for the standard.

It is also important that uncertainties in the source-to-detector distance cause minimal uncertainty in the calibration. This is achieved by making the active deposits as thin as possible so that energy loss in the source is small. With a thick source, variations in the calibration position could introduce enough intervening energy-absorbing air mass to remove a significant number of degraded energy alpha particles from detection. For a thin source, reasonable changes in the air mass between source and detector merely reduce the full energy of the monoenergetic alpha particles which arc nevertheless detected.

**Figure 11:**
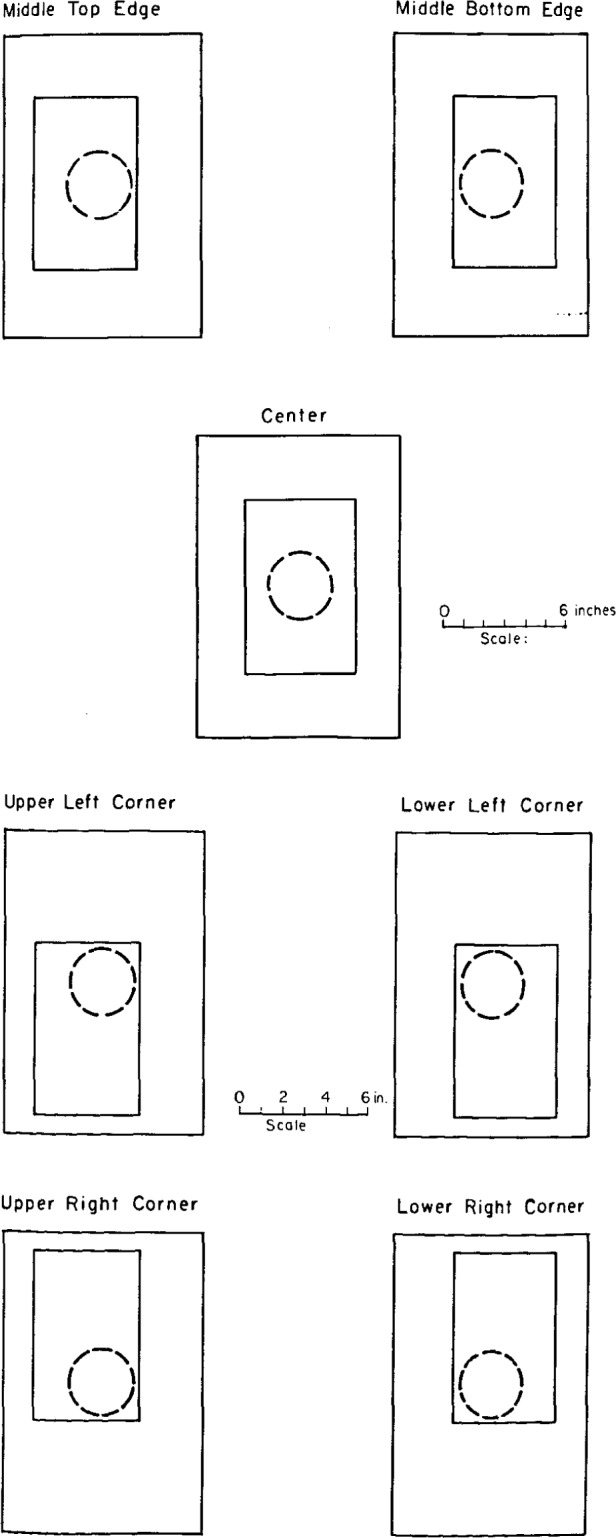
**a and b**-Source positions relative to the aperture of the external counter to test the homogeneity of the large-area sources, with results given in Table 2.

Experiments were performed to test both the homogeneity of the superficial activity and the response of the external detector and the AN/PDR-56F, as a function of source-to-detector distance. The homogeneity was tested using the external counter with no baffle (i.e., an aperture of 7 cm). Source positions relative to the aperture are given in figures 11a and 11b. Sources at each of the four activity concentration levels were tested with the results shown in table 2.

As seen in table 2, despite the fact that these are dried deposit sources, the maximum difference of any one of these readings is −6.3 percent from that of the center value. The maximum difference of average values relative to the center value is 1.021 or 2.1 percent. Presumably calibrations can be performed with an uncertainty from inhomogeneity on the order of 2 percent, since the calibration value is related to the average value.

**Figure 12:**
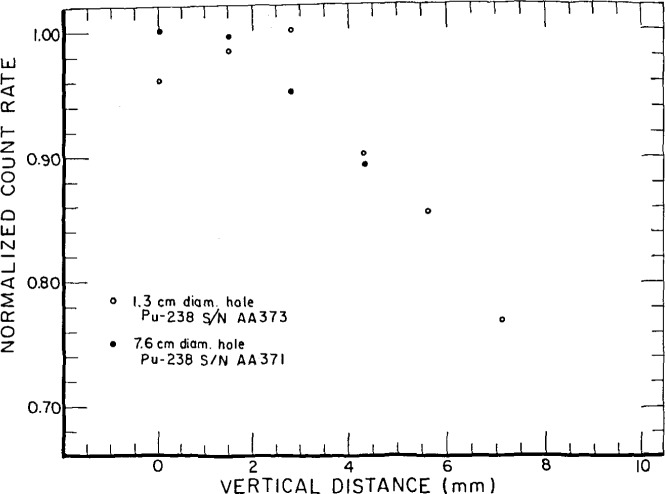
Count rate of a large-area ^238^Pu source normalized to the largest value measured with the external counter versus distance from the external counter. Zero distance corresponds to the source resting on the counter top (supported by the edges of the source). The distance from the active layer to the sensitive volume is 2 mm.

Figure 12 shows the response of the external detector for two apertures, 1.3 cm and 7.6 cm in diameter, normalized to the largest value. For the larger aperture, as the source approaches the detector, the count rate approaches an asymptotic value. For the smaller aperture, the effect of the spot character of the source manifests itself. The count rate goes through a maximum at a vertical distance of 3 mm, presumably because counts from adjacent spots are possible as the source moves away from the counter.

**Figure 13:**
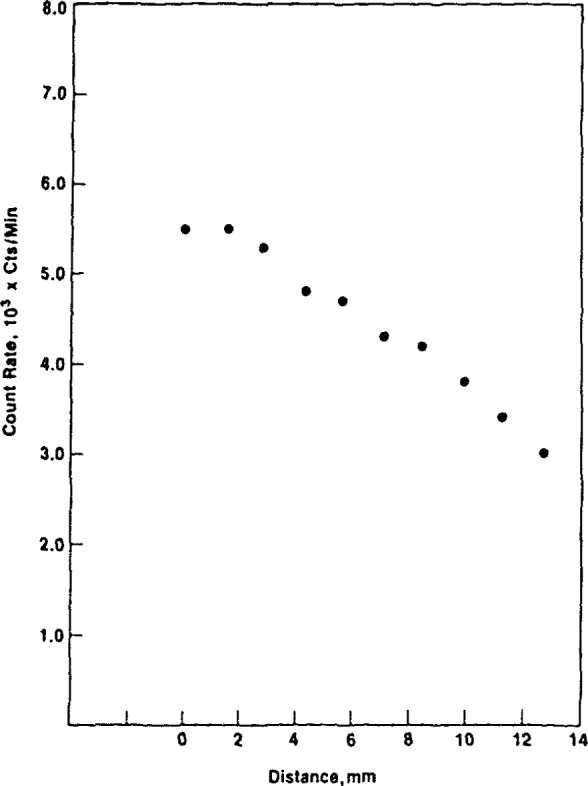
Count rate versus distance between a large area ^238^Pu source and the face of a DT-224B/PDR-56 probe. The zero distance corresponds to the condition where the probe and source are mounted in the UDM-7 jig. The active area is separated from the sensitive volume by 7-mm at “0” distance.

**Figure 14:**
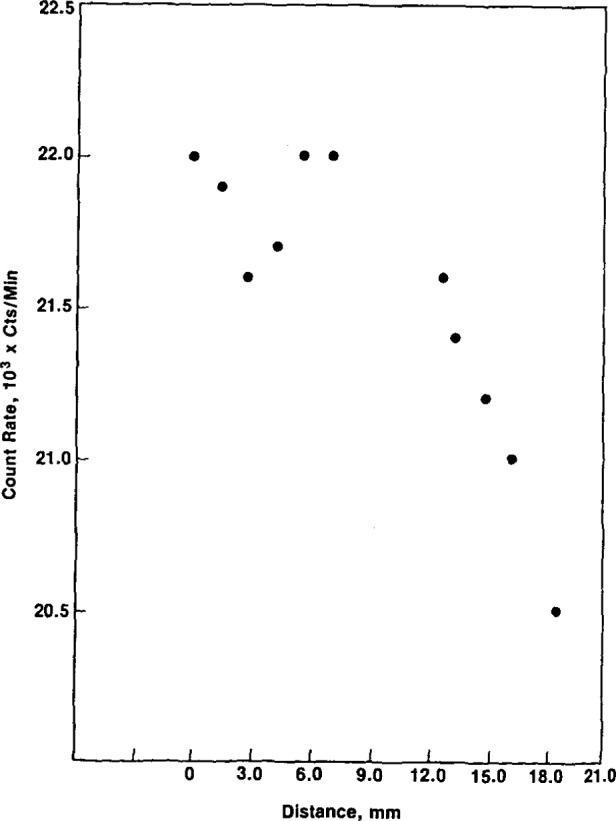
Count rate versus distance between a large area ^238^Pu source and an x-ray probe, DT-590/PDR-56F. The zero distance corresponds to the condition where the probe and source are mounted in the UDM-7 jig. The active area is separated from the sensitive volume by approximately 16-mm at “0” distance.

Another measure of the effect of vertical displacement of the source is shown in figures 13 and 14, measurements made with the AN/PDR-56F alpha-particle and x-ray probes, respectively. The alpha-particle probe is essentially a thin window plastic scintillation detector and the x-ray probe is a NaI(Tl) scintillation device. The x-ray data demonstrate much less variation as a function of distance.

Since the relative positioning of an unknown and a comparison standard source is within 1 mm, the uncertainty due to source positioning, at vertical distances in figure 12 between 0 and 1 mm, is less than a few tenths of one percent.

## 6. Calibration Procedure

The startup, source change, and shutdown procedures are given in the Appendix.

The radioactivity measurements of all four activity-level sources are based on 2*π*-internaI-counter measurements of the two lower-activity sources. The two higher-activity sources produce count rates in the internal counter beyond the dynamic range of the system. Consequently, the external counter was calibrated with the two previously calibrated low-activity sources, and the two higher-activity sources then compared in the same geometry.

**Figure 15:**
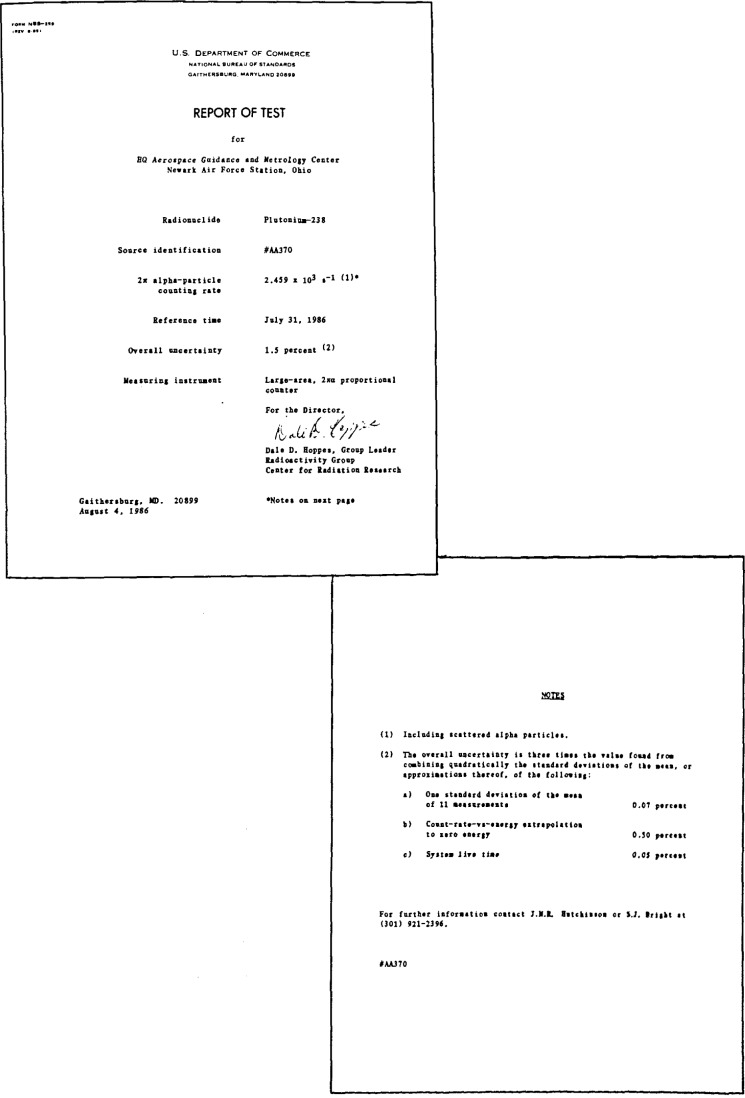
Typical certificate for source measured with the internal counter, the report test document above and the notes at right.

**Figure 16:**
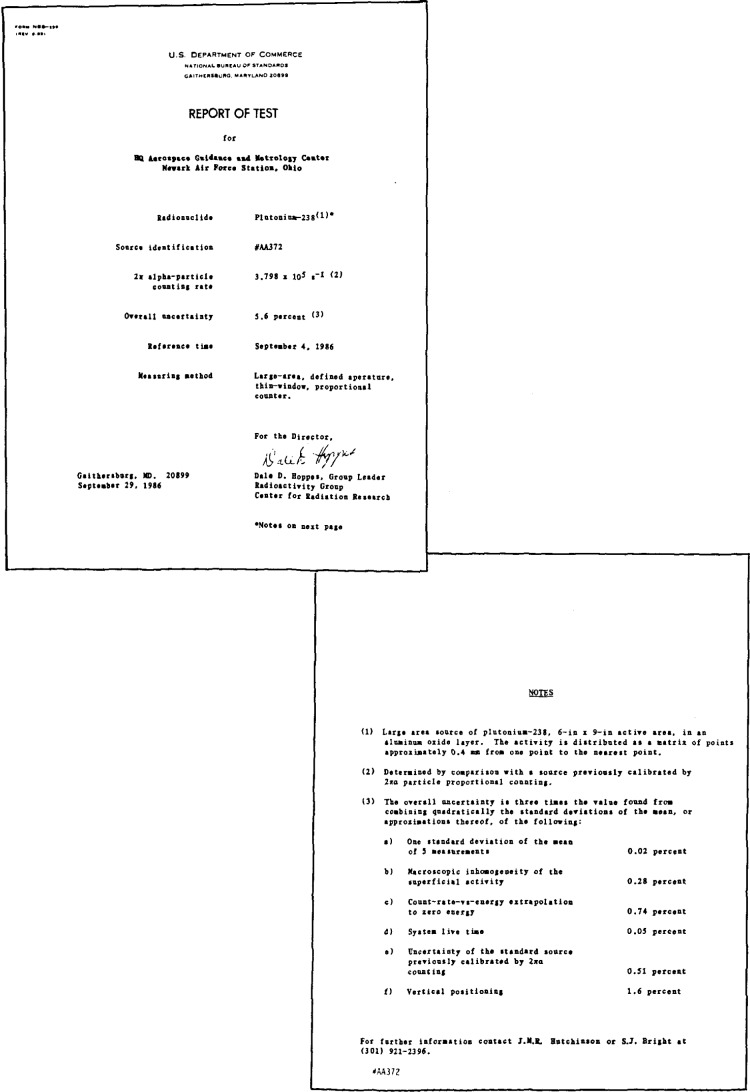
Typical certificate for source measured with the external counter, the report test document above and the notes at right.

The total 2*π*-alpha-particle rates thus measured are given in the certificate, typical examples of which are shown in figures 15 and 16, using respectively, the internal and external counters for the calibration.

The calibration procedure is as follows for counting with the 2*π* internal counter:

After the source has been introduced into the counter and the sensitive volume flushed until the pulses observed on the pulse-height analyzer have reached their maximum height, the counting procedure is initiated. Typically, the counting proceeds in the order: background, standard reference source, submitted source, standard reference source, background. The counting times are adjusted so that 10^6^ counts from the source are collected (corresponding to 0.1 percent statistical uncertainty). These counts are collected usually over five counting periods, with approximately 2×10^5^ counts per measurement.

The functioning of the instrument is checked by comparing the measurement results for the standard, corrected for decay, and the background with previous results.

Five pulse-height windows are set covering the lower end of the spectrum for the purpose of obtaining an extrapolation in the case where the total number of counts into 2*π* are required. The extrapolation is based on the approximation that the “true” tail of the spectrum can be represented as a flat horizontal line with a height corresponding to the spectrum minimum. Background is subtracted and the result, reduced to counts per second, is entered onto the calibration certificate. The MCA automatically corrects for system “dead time” if run on “live time”. The random uncertainty is taken as the internal standard deviation of the mean of the five repetitions with a total of 10^6^ counts. The corresponding random uncertainty would be 0.1 percent if other components of variation are all zero. A comparison of the internal standard deviation and, in this case, 0.1 percent is referred to as the “index of dispersion” test. Other uncertainties are estimated at the 1*σ* level and are added in quadrature. The overall uncertainty quoted in the certificate is three times the calculated combined uncertainty.

For comparison of a source previously calibrated in the internal counter with another (usually “hotter”) source using the “external” counter, a similar procedure is followed except that the known and unknown sources are counted alternately. The center of each source is placed over the aperture. As previously described (see table 2), uncertainties due to inhomogeneity average out to a large extent when count rates are referred to the central part of the source.

## Figures and Tables

**Table 1:** “Swipe” tests of Pu-238 large area sources.

Source No.	Count Time (min)	Alpha Count	Beta Count	Alpha DPM	Beta DPM
AA373	1	25	3	49 8	3.0
AA372	1	17	5	33.8	7.0
AA371	1	3	0	5.8	−3.0
AA370	1	4	0	7.8	−3.0

**Table 2:** Results of tests using four concentration levels and eight source positions. (Large Area External Detector #34152/Pu-238/No Baffle)

Area of Source	Count Rate Divided by Rate at the Center			
	Source No. AA371	Source No. AA370	Source No. AA841	Source No. AA840
Upper Left Corner	0.977	1.029	1.019	1.025
Lower Left Corner	0.977	1.021	1.013	1.024
Upper Right Corner	1.017	0.943	1.010	1.062
Lower Right Corner	1.033	0.937	1.016	1.021
Middle of Top	0.974	1.003	0.998	1.017
Middle of Bottom	1.003	0.980	0.967	0.977
Center	1.00	1.00	1.00	1.00
Average to Center	0.997	0.986	1.004	1.021

## Appendix

### 1. Operation of Large-Area Internal Counter

#### A. To Start

- Set up system (Connect H.V. supply, amplifier, preamplifier, H.V. interlock box and gas-flow indicator).
- Turn on electronics (NIM BIN), interlock box and power supply for the jack.
- With clamps released, lower the bottom plate of counter by placing switch in “down” position.
- Place source in center of counter, handling source with gloves to prevent contamination.
- Raise by placing switch in “up” position.
- After counter is closed, tighten clamps.
- Turn on gas (P-10, Argon-Methane) at main valve and adjust diaphragm control to flush rapidly for 10 minutes. NOTE: Do not exceed 2–3 psi (F=PA, where with a large area the force must be controlled by a small gas pressure). Turn on high voltage (+925 V) at start of flushing.
- After flushing, adjust regulator to produce a gentle gas flow.
- Connect amplifier to oscilloscope and observe pulses, usually with a coarse gain of 500, positive input, and bipolar output.
- Connect amplifier to analyzer, set summing regions, and collect data.

#### B. To Change Source

- Turn off H.V. and gas flow.
- Release clamps, lower counter, and change source.
- Raise counter and tighten clamps.
- Flush counter, turn on H.V.
- Observe pulses and collect data.

#### C. To Shut Down

- Turn off electronics (NIM BIN).
- Turn off gas (close main valve and release the diaphragm).
- Remove source or blank.
- Close counter.
- Turn off H.V. interlock box and power supply for the jack.
- Take smears of counter and immediate area and count at Health Physics.

### 2. Operation of Large-Area External Counter

#### A. To Start

- Set up system (Connect H.V. supply, amplifier, preamplifier, and gas-flow meter).
- Turn on electronics (NIM BIN).
- If desired, place appropriate baffle in detector face.
- Place source in a defined position on the detector, handling source with gloves to prevent contamination.
- Turn on gas (P-10, Argon-Methane) at main valve and adjust diaphragm control to 2–3 psi. Open regulator and flush rapidly for 10 minutes. Turn on high voltage (+95 V) at start of flushing.
- After flushing, adjust regulator to produce a gentle gas flow (~60 cc/min).
- Connect amplifier to oscilloscope and observe pulses, usually with a coarse gain of 500, positive input, and bipolar output.
- Connect amplifier to analyzer, set integrals, and collect data.

#### B. To Change Source

- Simply remove source and change to different source, placing it at a defined position.

#### C. To Change Baffle

- Turn off high voltage and gas flow.
- Remove source.
- Remove baffle, putting tweezers in two small holes in the edge of baffle and lifing up. (Be careful not to tear the mylar.)
- Replace with appropriate baffle or no baffle, if desired.
- Place source on detector in defined position.
- Turn on gas and again flush for 10 minutes. Turn on high voltage at the start of flushing.
- Adjust regulator for a gentle gas flow.
- Observe pulses and collect data.

#### D. To Shut Down

- Turn off electronics (NIM BIN).
- Turn off gas (close main valve and release the diaphragm).
- Remove source or blank.
- Cover the thin-window aperture with the solid baffle.
- Take smears of counter and immediate area and count at Health Physics.

